# Eosinophilic Gastrointestinal Diseases: Review and Update

**DOI:** 10.5402/2012/463689

**Published:** 2012-06-25

**Authors:** Mahreema Jawairia, Ghulamullah Shahzad, Paul Mustacchia

**Affiliations:** Department of Medicine, Nassau University Medical Center, 2201 Hempstead Turnpike, East Meadow, NY 11554, USA

## Abstract

Eosinophilic gastrointestinal disorders (EGIDs) are a progressively more frequent diverse group of intestinal diseases. The intention of this paper is to present the newest developments in the care of patients with EGIDs and to sum up a rising literature defining the clinical features and mechanistic elements of eosinophils and their intricate associations with the gastrointestinal tract. Clinicians ought to stay sensitive to EGIDs as a diagnostic likelihood for patients with general gastrointestinal symptoms. Further research is warranted to establish various methods leading to dysfunction coupled with eosinophilic gastrointestinal inflammation.

## 1. Introduction

Primary EGIDs (e.g., eosinophilic esophagitis, eosinophilic gastritis, eosinophilic gastroenteritis, eosinophilic enteritis, and eosinophilic colitis) are defined as disorders that primarily affect the gastrointestinal (GI) tract with eosinophil-rich inflammation in the absence of known causes for eosinophilia (e.g., drug reactions, parasitic infections, and malignancy). Even though the incidence of primary EGIDs has not been meticulously calculated, a miniepidemic of these diseases (especially EE) has been noted over the last decade. Eosinophils, a constitutive component of the columnar-lined gastrointestinal tract, play an essential role in allergic responses and parasitic infections. The tissue density of these cells also increases in a variety of conditions of uncertain etiology. With the exception of the esophageal squamous epithelium, in which no eosinophils are normally present, the population of normal eosinophils in the remainder of the luminal gut is not well defined [1]. There is limited information about normal eosinophil counts in the gastric mucosa. However, Lwin et al. [2] showed that the normal gastric eosinophilic counts are usually <38 eosinophils/mm. EGID is an uncommon gastrointestinal disease affecting adults and children. In 1937, Kaijser was the first to report a patient with eosinophilic gastroenteritis and, ever since, the disease is on the rise worldwide. The differential diagnosis of EGID includes parasitic infections, inflammatory bowel disease, connective tissue diseases, some malignancies, and adverse effects of drugs. It has been strongly associated with food allergies, and atopic diseases or a family history of allergies is elicited in about 70% of cases [3]. EGID can affect patients of any age but is more commonly seen in the third through fifth decades with a male predominance outside of the pediatric age group. Liacouras et al. [4]have found that 1% of their pediatric patients with GERD have EE, whereas Fox et al. [5] have reported that 6% of their patients with esophagitis have EE. EGIDs typically occur independent of peripheral blood eosinophil (>50% of the time) [4], indicating the potential significance of GI-specific mechanisms for regulating eosinophil levels. Evidence in support of the concept that EGIDs arise as a result of the interplay of genetic and environmental factors is accumulating. Markedly, a large percentage (approximately 10%) of patients with EGIDs have an immediate family member with an EGID [6]. The ensuing pathophysiological depiction in EGID is predominantly due to an immune-mediated mechanism where food-borne and aeroallergens are proven to have a crucial role [7]. Of the mediators associated with modifying eosinophil accumulation, IL-5 and the recently described subfamily of eotaxin chemokines are quite specific for eosinophils. Several studies [8] have identified IL-5 as a critical eosinophil growth factor and the eotaxins as critical tissue recruitment factors. Diagnosis of these disorders is dependent on the clinical presentation, endoscopic findings, and, most importantly, histological confirmation [9]. Guajardo et al. [10] reported that patients with EGIDs present with a variety of clinical problems, most commonly failure to thrive, abdominal pain, irritability, gastric dysmotility, vomiting, diarrhea, dysphagia, microcytic anemia, and hypoproteinemia. It is not unusual for the endoscopic appearance of the gastrointestinal tract to be normal, and as a result, microscopic assessment of biopsy samples is vital. According to Lee et al. [11], the disease frequently has patchy involvement, requiring the analysis of multiple endoscopic biopsy specimens from each intestinal segment.

## 2. Pathophysiology

**Figure 1:**
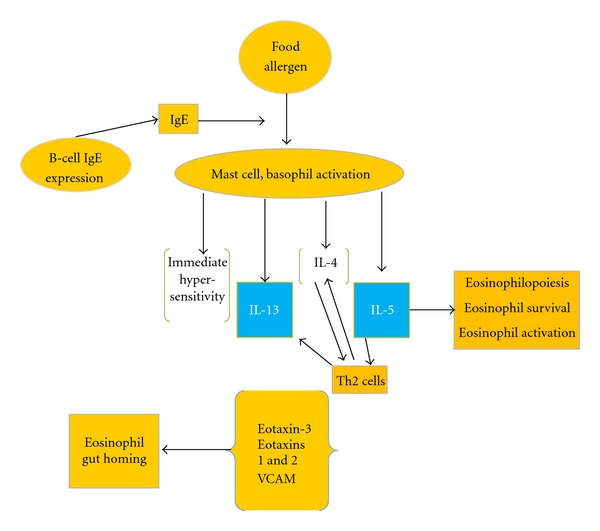
Pathophysiology in EGID.

Eosinophil aggregation in the gastrointestinal tract is a characteristic feature of various gastrointestinal conditions, including classic IgE-mediated food allergy [12], eosinophilic gastroenteritis [13], allergic colitis [14], eosinophilic esophagitis (EE) [15], inflammatory bowel disease (IBD) [16], and gastroesophageal reflux disease (GERD) [17]. The eosinophil is formed in the bone marrow, where it spends about 8 days maturing under the regulation of the transcription factors GATA-1, GATA-2, and c/EBP. These transcription factors provide “instructive” signals that cooperate with the “permissive” eosinophil growth factors IL-3, IL-5, and GM-CSF. IL-5 is the most specific to the eosinophil lineage and is responsible for the selective expansion of eosinophils and their release from the bone marrow. Eosinophils subsequently relocate into the peripheral circulation for 8 to 12 hours and finally traffic to specific tissues, predominantly the GI tract, where they reside for at least 1 week. Numerous inflammatory mediators have been implicated in regulating eosinophil accumulation, including IL-1, IL-3, IL-4, IL-5, IL-13, and GM-CSF and the chemokines RANTES, monocyte chemoattractant protein (MCP)3, MCP-4, macrophage inflammatory protein 1-alpha, and eotaxin 1, eotaxin 2, and eotaxin 3. IL-3 and GM-CSF, in association with IL-5, enhance eosinophil development, migration, and effector function, whereas IL-1, IL-4, IL-13, and TNF-*α* regulate eosinophil trafficking by promoting adhesive interactions with the endothelium. In collaboration with IL-5, chemokines and lipid mediators (platelet-activating factor and cysteinyl leukotriene [LT] C4) induce eosinophil trafficking by promoting chemoattraction.  Figure 1 depicts the pathophysiology in EGID. 

## 3. Role of IL-5, IL-13, and Eotaxin

Chemokines play a central role in eosinophilic migration and inflammation in both tissues and blood. In knockout mice experiments, Hogan et al. [18] challenged allergen-sensitized mice with oral allergen, in the form of enteric-coated beads resulting in marked eosinophil accumulation in the blood and small intestine in the control mice. Eotaxin is a chemokine, constitutively expressed in the gastrointestinal tract. In exotoxin-deficient mice, eosinophil recruitment into the mucosal lining was not seen with allergen stimulation, and these mice developed enhanced eosinophil accumulation in the blood compared with control mice [19]. Interestingly in IL-5-deficient mice, allergen challenge promoted partial eosinophil accumulation into the small intestine with a decline in peripheral eosinophil levels. These results established an IL-5-independent and eotaxin-dependent mechanism of accumulation of gastrointestinal eosinophils provide a molecular base which explains the dichotomy between peripheral blood and tissue eosinophilia [18]. Eotaxin (specifically eotaxin-3) is found overexpressed in patients with EE [10]. The cytokine IL-13 has an established role in eosinophilic infiltration in diseases such as asthma and other allergies [20, 21]. It has also been suggested that esophageal eosinophilic inflammation is mechanistically linked with pulmonary inflammation; this latter theory is based on the finding that repeated delivery of specific allergens or the TH2 cytokine IL-13 to the lung of mice induces experimental EE [22]. Mattes et al. [23] showed that mice deficient in both eotaxin 1 and IL-5 have a synergistic deficiency of allergen-induced lung eosinophilia and airway hyperreactivity, providing compelling evidence that both of these cytokines work together to elicit and regulate eosinophilia. There is now extensive preclinical data supporting a role for eotaxins in human disease, for example, there are markedly increased levels of eotaxin 1 mRNA in the lesions of patients with inflammatory bowel disease [24]. Eosinophils have been noted to be present at low levels in numerous tissues. When a large series of biopsy and autopsy specimens were analyzed, the only organs that confirmed tissue eosinophils were the gastrointestinal tract, spleen, lymph nodes, and thymus at significant levels [25]. Interestingly, eosinophil infiltrations were only associated with eosinophil degranulation in the gastrointestinal tract. Examination of eosinophils throughout the gastrointestinal tracts of conventional healthy mice has revealed that eosinophils are normally present in the lamina propria of the stomach, small intestine, cecum, and colon [26]. Unlike intestinal lymphocytes and mast cells, eosinophils are not normally present in Peyer's patches or intraepithelial locations, although they commonly infiltrate these regions in patients with EGIDs [27]. These facts suggest that eosinophils respond to different stimuli compared with other intestinal leukocytes. Indeed, constitutive expression of eotaxin 1 has been proven to provide the distinctive signal that promotes localization of eosinophils into the gastrointestinal tract at baseline.

## 4. Clinical Features

The frequency of specific symptoms varies depending on the intestinal segment involved, and there are no specific symptoms for diagnosing EGIDs. In patients with primary EE, frequently reported symptoms consist of vomiting, epigastric or chest pain, dysphagia, and respiratory problems [28, 29]. According to Kelly [30], the mucosal form of eosinophilic gastroenteritis (most common variant) is characterized by vomiting, abdominal pain (mimics acute appendicitis), diarrhea, melena, iron-deficiency anemia, malabsorption, protein-losing enteropathy, and failure to thrive. Infiltration of eosinophils in the muscularis layer leads to thickening of the bowel wall that may result in gastrointestinal obstructive symptoms mimicking pyloric stenosis or other causes of gastric outlet obstruction. The serosal form is seen in a minority of patients with eosinophilic gastroenteritis, and it is distinguished by exudative ascites with higher peripheral eosinophil counts compared with the other forms [31].

## 5. Endoscopic Features

A diagnostic evaluation for EGIDs should be performed on all patients with intractable symptoms, especially in individuals with a strong history of allergic diseases, peripheral blood eosinophilia, and/or a family history of EGIDs. On endoscopy, it is common to visualize linear creases oriented longitudinally (furrowing) in patients with EE [28]. However, in EE, endoscopic studies have shown strictures, mucosal rings, ulcerations, whitish papules, and polyps [14, 32]. In eosinophilic gastroenteritis, micronodules are noted on endoscopy, and these lesions often contain marked aggregates of lymphocytes and eosinophils [33]. On endoscopic examination of patients with eosinophilic colitis, patchy erythema, loss of vascularity, and lymphonodular hyperplasia are seen typically localized to the rectum but may extend to the entire colon [34]. 

## 6. Histopathology

The diagnosis of an EGID is dependent on the microscopic evaluation of endoscopic biopsy samples, with careful attention to the quantity, location, and characteristics of the eosinophilic inflammation. It is not uncommon for the endoscopic appearance of the gastrointestinal tract to be normal, and thus microscopic evaluation of biopsy samples is essential. The number and location of eosinophils are useful when trying to differentiate EE from GERD. Up to 7 eosinophils/hpf (400x) is most indicative of GERD, 7 to 20 to 24 eosinophils/hpf likely represents a combination of GERD and food allergy, and more than 20 to 24 eosinophils/hpf is characteristic of EE [35]. Histologic analysis of the small bowel from patients with eosinophilic gastroenteritis reveals extracellular deposition of eosinophil granule constituents, and indeed, extracellular major basic protein and eosinophil cationic protein are immunohistochemically detectable at increased levels [36]. Although there are focal aggregates of eosinophils in the lamina propria, crypt epithelium, and muscularis mucosa and, occasionally, the presence of multinucleated giant cells in the submucosa, histologic examination often reveals that the overall architecture of the mucosa is well preserved in patients with eosinophilic colitis [8].

## 7. Allergic Association

Food allergy can be defined as an abnormal immunological response to food proteins that can cause an adverse clinical reaction [37]. The most common foods reported in EGID are eggs, milk, and fish, and there are many other food particles related to development of EGID [38]. Rothenberg et al. [39] showed their recent models of EGID that supported a potential allergic cause for these disorders. Even though there is a common finding of food-specific IgE in patients with EGIDs, food-induced anaphylactic responses only occur in a minority of patients [36, 40]. Therefore, EGIDs have features that fall between pure IgE-mediated food allergy and cellular-mediated hypersensitivity disorders (e.g., celiac disease) [41]. In EE, the majority of patients have evidence of food and aeroallergen hypersensitivity, as defined by skin prick tests, RASTs, or both; however, only a minority has a history of food anaphylaxis [21]. In one pediatric study, children with EE were found to have more than 60% with food allergies [42]. In one large retrospective analysis, children with diagnosis of EE have more intense symptoms during summer or fall than in winter [43]. Environmental allergens and pollens are associated with EE. Fogg et al. reported a case of 21-year-old female presenting with diagnosis of EE and seasonal variation in biopsy-proven eosinophils count [44]. Primary eosinophilic enteritis, gastritis, and gastroenteritis are also known as idiopathic or allergic gastroenteropathy, whereas the familial form has not been well characterized but is seen in about 10% of patients [23].

## 8. Treatment Options

Dietary elimination, systemic and topical corticosteroids, leukotriene receptor antagonists, and most recently investigational biologic therapies have been used to treat EGID. Conversely, the best single possible therapy has not yet been defined. The available literature for possible treatment is either with case series or a very small clinical trial with poor clinical outcomes.

### 8.1. Diet Therapy

 A trial of specific food antigen and aeroallergen avoidance is often indicated for patients with EGID. It has been shown that an elemental diet often improves symptoms and lowers the number of eosinophils in the esophageal biopsy specimens in patients with primary EE (allergic or nonallergic subtypes) [45]. A large randomized prospective study was conducted in patients with a diagnosis of EE that were tested for food allergy with skin prick or atopy with a skin patch. In those subjects with a positive test result, a restricted diet was applied for 4–8 weeks, and a significant histological improvement of esophageal inflammation was documented in more than 70% of the study population [46]. Eliminating the dietary intake of the foods implicated by skin prick testing (or RASTs) has unpredictable effects, but complete resolution is generally attained with amino acid-based elemental diets in patients with EGE [47]. In EC, on removal of the offending protein trigger in the diet, the gross blood in the stools generally resolves within 72 hours, but occult blood loss might endure longer [48]. The recurrence of symptoms with reintroduction of nonelemental foods is a major drawback in this study group. Moreover, these approaches have significant risk of nutritional deprivation and can lead to significant psychological burden on patients and their families [49]. Hence, a registered dietician may be helpful in the management of these patients [37].

### 8.2. Steroids

Glucocorticoids (systemic [14] or topical [48]) have also been used with suitable results. Systemic steroids are utilized for acute exacerbations, whereas topical steroids are used to provide long-term relief in cases in which diet restriction is not feasible or has failed to improve the disease in EGID [16]. In a prospective cohort series, all patients were treated with oral steroids for 4 weeks and were followed with a clinical and histological evaluation before and after treatment. Patients in steroid group had a histological improvement by decrease in eosinophilic count on biopsy and 65% of 20 patients had complete resolution of symptoms. In spite of significant early improvement, majority of the patients relapsed after the withdrawal of steroid therapy, and their role as a long-term management strategy is still unclear [50].

### 8.3. Montelukast

Montelukast selectively blocks the D4 receptor of cysteinyl leukotrienes present in eosinophils. All cysteinyl leukotrienes are arachidonic acid-derived inflammatory mediators [51] which are important in asthma [52]. The actions of leukotrienes include eosinophil attraction and migration, powerful constriction of smooth muscle, airway edema, mucous hypersecretion, and reduction in ciliary motility [53, 54]. They are predominantly released by eosinophils, basophils, and mast cells [55, 56]. By blocking the D4 receptor, the inflammatory action of the eosinophil cell is reduced. Treatment success with montelukast is similar to steroids, with recurrence of disease upon withdrawal. There have been few papers demonstrating symptomatic improvement of EE with montelukast [57]. However, when cysteinyl leukotriene levels were examined in biopsies from patients with EGID, the only statistically significant increase in leukotrienes was found in patients with eosinophilic gastroenteritis [58].

## 9. New Biologic Treatments

### 9.1. Anti-IL5 Therapy

 Based on the function of IL-5 in the development, differentiation, mobilization, activation, and survival of eosinophils [59] and preclinical studies in mice [22, 60], therapeutics targeting IL-5 are obvious candidates for trials in patients with EGIDs [61]. A pilot study treating 4 patients with eosinophilic gastroenteritis with a single dose of humanized anti-IL-5 monoclonal antibody (SCH55700) resulted in a decrease in peripheral eosinophilia (mean decrease of 70%) and tissue eosinophilia (50–70% decrease in 3 of 4 subjects), but minimal improvement in symptoms [62]. Interestingly, one patient had a 43% increase in gastrointestinal eosinophil counts 4 weeks after treatment. In addition, 7-8 weeks after treatment, 2 of 4 subjects had a significant increase in peripheral eosinophil counts and worsening of their baseline symptoms [63]. The first reported patient with eosinophilic esophagitis treated with the humanized anti-IL-5 antibody, mepolizumab, was an 18-year male with a lifelong history of dysphagia, persistent vomiting, and a severe stricture on endoscopy, who failed to respond to dietary restrictions or topical or systemic corticosteroids [64]. As part of an open-label trial, he received 3 doses of mepolizumab at 4-week intervals. On this regimen, there was a >10-fold decrease in the mean number of tissue eosinophils, diminished gross inflammation and stricture on endoscopy, improvement in symptoms with cessation of vomiting, and advancement of his diet to solids. Larger randomized, controlled trials are needed to further clarify the efficacy and safety of this therapy and to clarify its role in the long-term management of patients with EGIDs, including use in patients at earlier stages of disease and with less severe presentations. The finding of elevated IL-5 levels after treatment with mepolizumab in a recent paper [65], and the previous paper of rebound eosinophilia after cessation of therapy, also raises questions about long-term dosing strategies if long-term therapy is required. If future therapeutic regimens use anti-IL-5 therapy, unless specific “responders” to anti-IL-5 can be identified, targeting of additional mediators would likely be needed as well.

### 9.2. Anti-IgE Therapy

 Patients with EGIDs frequently have multiple food-specific IgE levels detectable by skin testing and/or *in vitro* testing and, particularly with EE, respond to dietary restrictions. In addition, mast cell numbers are increased in esophageal biopsies from patients with EGIDs [66], activated mast cells are identified by electron microscopy of esophageal biopsies of patients with EE [67], and an association between mast cell numbers and degree of esophageal eosinophilia and epithelial hyperplasia has been described [7]. Omalizumab is a humanized therapeutic monoclonal antibody that binds to IgE, thus preventing IgE from activating mast cells and basophils and decreasing the concentration of high-affinity IgE receptors on these cells. Omalizumab is used in severe atopic asthma that is not controlled by maximal medical therapy using conventional asthma drugs. Currently, omalizumab is the only clinically available anti-IgE therapeutic. Anti-IgE lowers eosinophil counts in both the blood and lungs of asthmatic subjects [68], which suggested that anti-IgE could be a potential therapeutic approach in EGIDs. Foroughi et al. [69] examined the potential efficacy of omalizumab in a 16-week open label study of 9 subjects with allergic eosinophilic gastroenteritis. Omalizumab was associated with a 35–45% drop in peripheral blood eosinophil count as well as larger-magnitude 60–70% decreases in duodenal and antral eosinophils. In contrast, esophageal eosinophils were modestly increased during the study, providing further support that EE and EG are distinct clinical entities with different pathophysiologic features. Omalizumab is dosed in proportion to a patient's serum IgE, and its efficacy is directly related to its ability to lower free IgE [70]. Therefore, omalizumab is unlikely to be effective in EGID patients with a serum IgE above 700 kIU/L. Accordingly, second-generation anti-IgE therapeutics with greater capacity to block IgE may have greater efficacy in EGIDs.

### 9.3. Anti-TNF Therapy

 Anti-TNF therapies are currently approved for the treatment of inflammatory bowel disease, rheumatoid and psoriatic arthritis, ankylosing spondylitis, and plaque psoriasis. Trials of anti-TNF therapy (etanercept) in patients with refractory asthma and elevated TNF expression in monocytes, a small subset of asthmatics, have also been promising, although of benefit only to a small subset of patients [71]. Since esophageal epithelial cells in patients with active EE express significantly higher levels of TNF relative to controls [66], a recent paper describes the results of treatment of 3 adults with severe, corticosteroid-dependent EE [72]. The 3 patients were taken off all treatments for EE during a 4-week run-in period and then treated with 2 doses of infliximab (5 mg/kg) spaced 2 weeks apart as monotherapy. With this treatment, 2 patients had very mild improvement in symptoms, while 1 had worsening symptoms. There was no significant decrease in eosinophils or mast cells in the 2 responders, and there was a modest decrease in TNF expression in esophageal epithelial cells. As a result, this approach does not appear promising for monotherapy of EE; however, use at higher doses and/or for longer periods of time may be necessary to demonstrate efficacy.

## 10. Discussion

**Figure 2:**
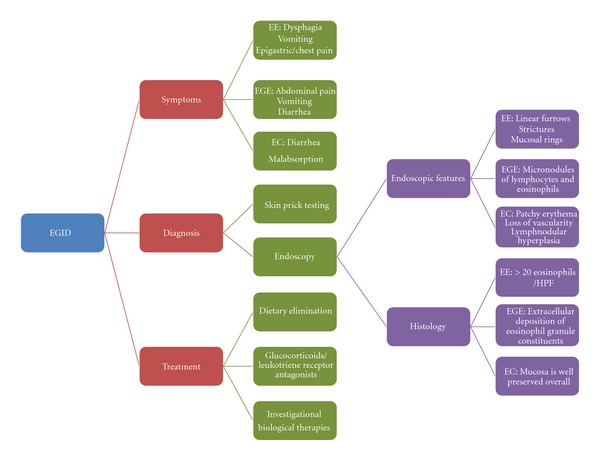
EGID in a nutshell.

Primary eosinophilic gastrointestinal disorders are defined as disorders that selectively affect the gastrointestinal tract with eosinophil-rich inflammation in the absence of known causes for eosinophilia (e.g., drug reactions, parasitic infections, and malignancy). These disorders include eosinophilic esophagitis, eosinophilic gastritis, eosinophilic gastroenteritis, eosinophilic enteritis, and eosinophilic colitis and are occurring with increasing frequency (Figure 2). Significant progress has been made in explaining that eosinophils are integral members of the gastrointestinal mucosal immune system and that eosinophilic gastrointestinal disorders are primarily polygenic allergic disorders that involve mechanisms that fall between pure IgE-mediated and delayed TH2-type responses [16]. Although considerable advances are made in the understanding of human pathogenesis, the next challenge will be to understand the clear molecular mechanisms involved in EGID disease expansion. Future studies may provide us with new biomarker to differentiate EGID from other gastrointestinal disorders. The diagnostic criterion of EGID based on endoscopic biopsy is still debatable and needs more refinement. The sites of the biopsy depending on the gross picture of the gastrointestinal mucosa have not been established. Although some treatments are effective in EGID, the molecular mechanisms involved in the remission have still not been established. The development of *in vitro* and *in vivo* models may help to dissect out the molecular mechanisms involved in remission or resistance to therapy. The overall goal is being able to molecularly classify patients as a function of their predicted response to treatment. In summary, EGID appears to be increasing worldwide. The increase in prevalence suggests a need for more definitive diagnostic criteria and treatment. The pathophysiology of EGID suggests the role of certain food or aeroallergens in a genetically susceptible individual. A multidisciplinary team involving a primary care physician, endoscopist, nutritionist, and allergist immunologist might be a superior approach to deal with this disease. More definitive research is indicated to further elucidate the role of eosinophilic mediators in order to plan treatment options such as diet and anti-inflammatory therapy.
